# Deciphering the Mechanism by Which Carbon Dioxide Extends the Shelf Life of Raw Milk: A Microbiomics- and Metabolomics-Based Approach

**DOI:** 10.3390/molecules29020329

**Published:** 2024-01-09

**Authors:** Anran Zheng, Chaokun Wei, Jun Liu, Ningxia Bu, Dunhua Liu

**Affiliations:** 1 School of Animal Science and Technology, Ningxia University, Yinchuan 750021, China; zhenganran17@163.com (A.Z.);; 2 School of Food Science and Engineering, Ningxia University, Yinchuan 750021, China; 3 School of Life Sciences, Hubei Normmal University, Huangshi 435002, China; dove_lj@126.com

**Keywords:** raw milk, CO_2_ treatment, microbiomics, metabolomics

## Abstract

Microbial community succession in raw milk determines its quality and storage period. In this study, carbon dioxide (CO_2_) at 2000 ppm was used to treat raw milk to investigate the mechanism of extending the shelf life of raw milk by CO_2_ treatment from the viewpoint of microbial colonies and metabolites. The results showed that the shelf life of CO_2_-treated raw milk was extended to 16 days at 4 °C, while that of the control raw milk was only 6 days. Microbiomics analysis identified 221 amplicon sequence variants (ASVs) in raw milk, and the alpha diversity of microbial communities increased (*p* < 0.05) with the extension of storage time. Among them, *Pseudomonas*, *Actinobacteria* and *Serratia* were the major microbial genera responsible for the deterioration of raw milk, with a percentage of 85.7%. A combined metagenomics and metabolomics analysis revealed that microorganisms altered the levels of metabolites, such as pyruvic acid, glutamic acid, 5′-cmp, arginine, 2-propenoic acid and phenylalanine, in the raw milk through metabolic activities, such as ABC transporters, pyrimidine metabolism, arginine and proline metabolism and phenylalanine metabolism, and reduced the shelf life of raw milk. CO_2_ treatment prolonged the shelf life of raw milk by inhibiting the growth of Gram-negative aerobic bacteria, such as *Acinetobacter guillouiae*, *Pseudomonas fluorescens*, *Serratia liquefaciens* and *Pseudomonas simiae*.

## 1. Introduction

Raw milk is commonly produced intensively due to its collection from dispersed farms and subsequent transportation to processing plants for uniform treatment. The stages of the collection, transportation, and waiting for processing of raw milk last for a significant duration (several hours), imposing heightened demands on its quality [1,2,3]. The diversity of microbial communities in raw milk is usually considered as one of the key factors affecting milk quality, because udder surfaces, milking instruments, containers and environment are potential sources of microorganisms, which leads to microorganisms being inevitable in raw milk [4]. Given its nutrient-rich composition, raw milk provides an optimal environment and a nutrient matrix that facilitate rapid microbial reproduction [5]. Consequently, this can result in undesirable outcomes, such as off-flavors, sourness, discoloration, and a loss of the characteristic freshness associated with milk [1]. Additionally, microorganisms break down and consume essential nutrients present in milk, including fats, proteins and vitamins. This process may lead to a reduction in the nutrient content of raw milk, thereby impacting the nutritional value of the final dairy product [6]. In particular, psychrophilic and psychrotolerant bacteria (such as *Pseudomonas* and Enterobacteriaceae) that grow rapidly at low temperatures are considered the most abundant and harmful spoilage bacteria in raw milk [7]. The heat-resistant proteases and lipases produced during the heat treatment process before processing cannot be completely inactivated, leading to a decrease in the yield and quality of dairy products, such as high-temperature sterilized milk, cheese, yogurt, and ice cream [8]. To ensure the provision of high-quality and safe dairy products while prioritizing consumer health and satisfaction, it is imperative to implement effective control measures aimed at mitigating or eliminating microbial hazards inherent in raw milk [2,5].

For the past few years, researchers have explored the use of high pressure to inactivate microorganisms in raw milk while preserving its nutritional quality. However, due to the complexity of high-pressure equipment and limited treatment efficiency, it is not suitable for large-scale implementation [9]. Non-thermal plasma treatment offers an alternative approach to generating reactive oxygen and nitrogen species that can penetrate microorganisms and inhibit or kill them. However, these oxides can also cause the oxidation of raw milk, leading to a decline in its quality as a complex emulsion system [10]. Conventional sterilization methods may destabilize raw milk by affecting its composition and structure. To overcome these challenges, alternative technologies, such as gas conditioning or non-thermal treatments, are being investigated to minimize adverse effects on raw milk while preserving its nutritional value and sensory properties [11].

CO_2_ has been used for fresh milk storage and preservation because it does not affect the nutritional content or flavor compared to conventional heat treatments. Also, CO_2_ is a natural compound that does not leave harmful residues in the product or the environment [12,13]. CO_2_ has been used for food preservation. When CO_2_ is dissolved in milk, it forms carbonic acid, which lowers the pH of the milk. Many bacteria, including those that cause spoilage, cannot survive or grow effectively in this more acidic environment [14,15]. In addition, by replacing oxygen with CO_2_ in the storage container, packaging or food systems (a technique known as modified atmosphere packaging), the growth of aerobic bacteria and fungi is inhibited. These microorganisms require oxygen to grow, thus removing or reducing oxygen levels can help extend the shelf life of raw milk [16].

In view of the complexity of the raw milk system and microbial community succession, the metabolic pattern of CO_2_ treatment on raw milk and microorganisms was analyzed by using macro-genome and metabolome, and then the molecular mechanism by which CO_2_ prolongs the shelf life of raw milk was clarified. The results of the study provide a theoretical basis for the application of CO_2_ in raw milk.

## 2. Results

### 2.1. Physicochemical Characteristics of Raw Milk

**Figure 1:**
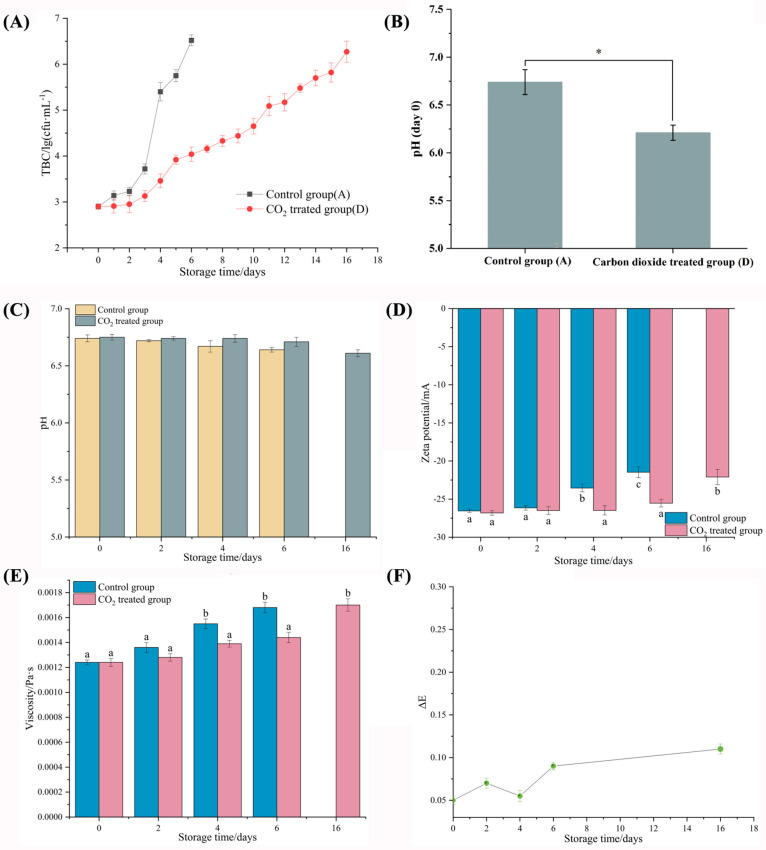
(**A**) Total bacterial counts of raw milk stored at 4 °C. (**B**) Effect of CO_2_ treatment on raw milk pH. (**C**) Effect of CO_2_ treatment after degassing on the pH of raw milk. (**D**) Effect of CO_2_ treatment after degassing on the Zeta potential of raw milk. (**E**) Effect of CO_2_ treatment after degassing on the viscosity of raw milk. (**F**) Effect of CO_2_ treatment on the color difference of raw milk. (Different lowercase letters and * indicate a significant difference, *p* < 0.05).

Total bacterial counts (TBC) were utilized to assess the storage period of raw milk (Figure 1A). The control group was identified as spoilage on day 6, with the TBC greater than 6 lg (cfu/mL). In contrast, the TBC of the CO_2_-treated group was only 4.03 lg (cfu/mL) on day 6 and did not exceed 6 lg (cfu/mL) until day 16, indicating that the CO_2_ treatment prolonged the storage time of raw milk by significantly inhibiting the growth of the bacteria. It is noteworthy that the TBC of the control raw milk increased steadily during the first 3 days, and showed a logarithmic increase on day 4, suggesting that day 4 of refrigeration may be a critical time point for changes in the quality of raw milk. Therefore, this study selected samples from days 0, 4 and 6 for metagenomic and metabolomic analysis to explore the mechanism of CO_2_ prolonging the cold storage period of raw milk.

In addition, CO_2_ at 2000 ppm significantly reduced the pH of raw milk (*p* < 0.05) (Figure 1B). But interestingly, when they were degassed, the pH value increased to exceed that of the control group until day 16 (pH 6.61, similar to the pH value (6.64) on day 6 of the control group) (Figure 1C). The zeta potential, viscosity and color in the samples were then analyzed. As shown in Figure 1D–E, the zeta potential and viscosity of both the control and treated samples tended to increase during the refrigeration period, indicating a decrease in the stability of the emulsion system. However, the rate of decrease in both treatment groups was slower than that of the control group during the same refrigeration time, indicating that the addition of CO_2_ increases the stability of raw milk. The color difference (ΔE) reflected the magnitude of the color change, and when ΔE > 3.8, the raw milk showed a color difference visible to the naked eye [17]. Throughout the refrigeration period, the ΔE values between the CO_2_ treated group and the control group ranged from 0.05 to 0.11 (Figure 1F), indicating that CO_2_ treatment had almost no effect on the color of the raw milk.

### 2.2. Microbial Diversity of Raw Milk

**Figure 2:**
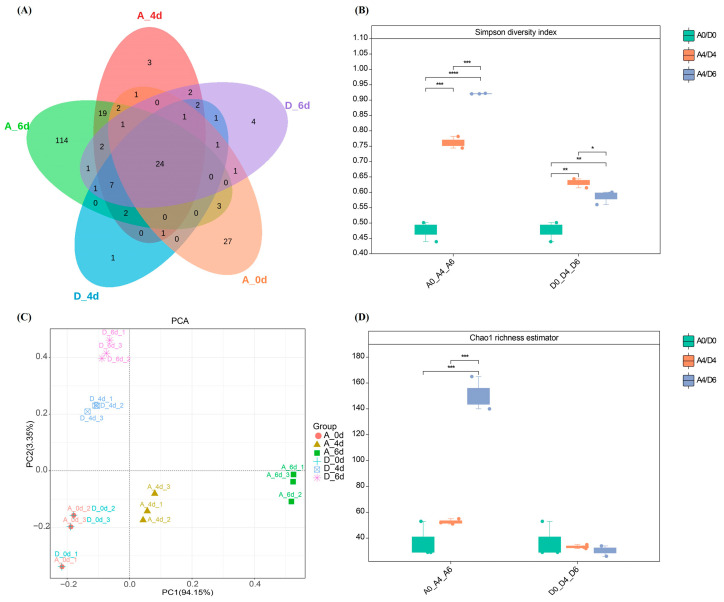
Microbial diversity of raw milk from the control and CO_2_-treated groups. (**A**) Number of common and unique microbial taxa in raw milk from both groups. (**B**) Simpson’s index to assess the alpha diversity (genus level) of microbial communities in the two groups. (**C**) Principal component analysis (PCA) to assess β-diversity (genus level). (**D**) Chao1 index to assess the α-diversity (genus level) of the two microbial communities. (A0, sample of control group at 0 d; D0, sample of CO_2_-treated group at 0 d, and so on.) (Significant differences were recorded by * *p* < 0.05, ** *p* < 0.01, *** *p* < 0.001).

Analysis of the composition of the biome in the raw milk using the Illumina sequencing platform showed that there were 221 ASVs in the raw milk. The control group contained a total of 189 ASVs, 136 of which were unique to that group. Of these, group A0 had 62 ASVs, 27 of which were unique to that group; group A4 had 67 ASVs, three of which were unique to that group; group A6 had 176 ASVs, 114 of which were unique to that group; and the CO_2_-treated group contained a total of 85 ASVs, five of which were unique to that group. Of these, the day 4 group had 41 ASVs, one of which was unique to that group; and the day 6 group had 48 ASVs, four of which were unique to that group (Figure 2A). These findings suggested that CO_2_ treatment reduced the microbial diversity compared to the control group. The community richness of raw milk microbiota (Simpson diversity index and Chao1 richness estimator) also showed that CO_2_ treatment reduced raw milk microbial α-diversity (Figure 2B,D). Principal component analysis (PCA) showed that the samples from day 0 clustered in the third quadrant. With the extension of refrigeration time, the control group continued to move towards the fourth quadrant along the x-axis direction, while the CO_2_ treated group continued to move towards the second quadrant along the y-axis direction. There was a significant separation between the two groups, confirming a significant difference in microbial β-diversity between the CO_2_ treated raw milk and the control group (Figure 2C).

### 2.3. Microbiological Composition of Raw Milk

**Figure 3:**
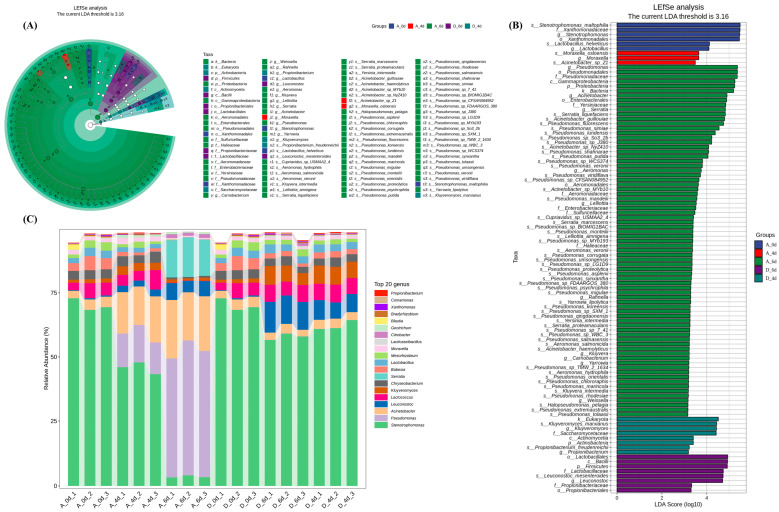
Microbial community structure of raw milk from the control and CO_2_ treated groups. (**A**) Linear discriminant analysis effect size (LEfSe) species branching diagram. The taxonomic branching diagram shows the taxonomic hierarchy of the major taxonomic units in the sample community from phylum to species (from inner circle to outer circle, phylum, order, order, family, genus and species), with node sizes corresponding to the average relative abundance of the taxonomic unit; hollow nodes represent taxonomic units with insignificant intergroup differences, while nodes of other colors indicate that these taxonomic units exhibit significant intergroup differences and are more abundant in the sample than in the group represented by the color. Higher abundance in the sample. Letters identify the names of taxonomic units with significant intergroup differences. (**B**) Histogram of LEfSe. Vertical coordinates show taxonomic units with significant differences between groups, and horizontal coordinates visualize the logarithmic scores of LDA analysis for each taxonomic unit in a bar chart. Taxonomic units are sorted by the size of the score to characterize their specificity within the sample grouping. Longer lengths indicate more significant differences for that taxonomic unit, and the color of the bar indicates the sample subgroup with the highest abundance corresponding to that taxonomic unit. (**C**) Taxonomic analysis of bacteria at the genus level. Horizontal coordinates are arranged according to the sample name, each bar represents one sample and is color-coded to distinguish each taxonomic unit, vertical coordinates represent the relative abundance of each taxonomic unit, the longer the bar, the higher the relative abundance of that taxonomic unit in the corresponding sample.(A_0d, sample of control group at 0 d, D_0d, sample of CO_2_ treated group at 0 d, and so on).

The differences in microbial community composition between the two groups were analyzed by nonparametric Kruskal–Wallis and Wilcoxon rank-sum tests, combined with linear discriminant analysis (LDA) and effect size. A total of 100 microbial markers with LDA > 2 and *p* < 0.05 were identified at different levels. Notably, Figure 2A and Figure 3A showed consistent differences in α- and β-diversity indices, especially in the A6 microbial community, the number of biomarkers was much higher than the D6 group, suggesting that the CO_2_ treatment inhibited microbial reproduction. As shown in Figure 3B, the initial microbial communities significantly enriched in raw milk included *s_Stenotrophomonas_maltophilia*, *g_Stenotrophomonas*, *f_Xanthomonadaceae*, *s_Lactobacillus_helveticus*, *g_Lactobacillus_helveticus* and *g_Lactobacillus*. When raw milk spoiled, the microorganisms significantly enriched in group A6 were mainly *k_Bacteria*, *p_Proteobacteria*, *c_Gammaproteobacteria*, *f_Pseudomonadaceae*, *o_Pseudomonas* and *g_Pseudomonas*, suggesting that these may be the key microorganisms responsible for the deterioration of raw milk quality. The significantly enriched in the D6 group treated with CO_2_ were *p_Firmicutes*, *c_Bacilli*, *o_Lactobacillales*, *f_Lactobacillaceae*, *g_Leuconostoc* and *s_Leuconostoc_mesenteroides*. The sequences were annotated at the genus level for classification, and the results were shown in Figure 3C. *Stenotrophomonas* accounted for 70.2% of the initial flora of raw milk (A0/D0), and the abundance gradually decreased with the extension of the cold storage time, but still accounted for the highest proportion in groups A4, D4 and D6 (45.7%, 62.1% and 57.9%, respectively). CO_2_ treatment had a significant effect on the microbial species at the genus level, with *Pseudomonas*, *Actinobacteria* and *Serratia* significantly increasing to 49.2%, 20.9% and 14.6% in the control group at day 6, but only to 0.09%, 3.0% and 0.03% in the D6 group.

### 2.4. Metabolites

#### 2.4.1. Data Quality Control

**Figure 4:**
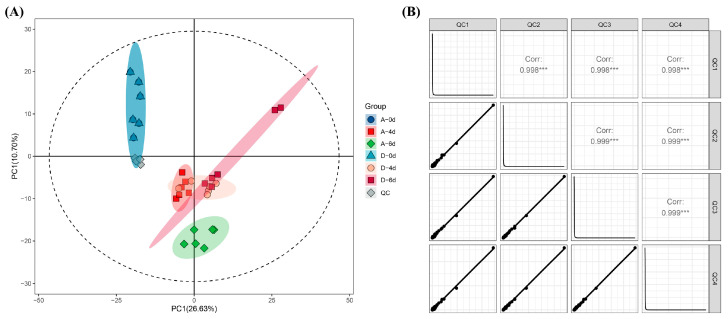
(**A**) PCA score plots for raw milk metabolite and quality control (QC) sample datasets. (**B**) Correlation plot for QC samples. (*** Indicating significant correlation, *p* < 0.001).

Microorganisms use raw milk as a substrate for reproduction and, in addition to changes in microbial numbers and species, alter the raw milk metabolite system through their own metabolic activities. The study aimed to assess how CO_2_ treatment affects raw milk metabolite evolution by influencing microbial succession and, consequently, raw milk quality. LC–MS-based untargeted metabolomics identified a total of 1234 metabolites in six batches of raw milk. PCA was initially used as a way to explore the structure of the data and assess its distribution and metabolite variability in PC space. Six raw milk samples and four QC samples were used as the dataset. First, according to Figure 4A,B, the distributions of the four QC samples in the PCA model overlapped almost completely, and the correlation coefficients were all greater than 0.998, suggesting that mass spectrometry detection was reliable. As shown in Figure 4A, A0 and D0 sample metabolites had good similarity, indicating that CO_2_ did not affect the quality of raw milk. A4, A6, D4 and D6 were distinguished from the 0-day raw milk group. However, the A4, D4 and D6 metabolites partially overlapped in the whole, indicating that it is not feasible to use PCA unsupervised modeling alone to distinguish the raw milk metabolite groups.

#### 2.4.2. Overview of Differences between Samples

**Figure 5:**
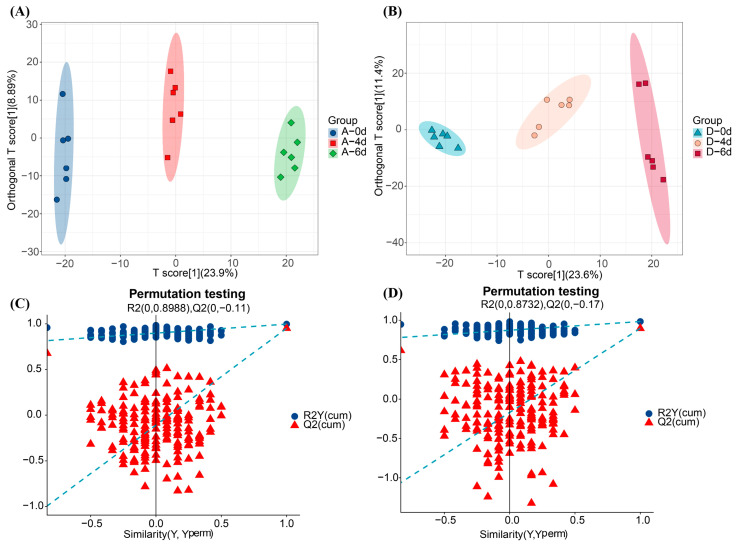
(**A**) OPLS-DA of the metabolite dataset of the control group. (**B**) OPLS-DA of the metabolite dataset of the CO_2_-treated group. (**C**) Alignment test results of the OPLS-DA of the control group. (**D**) Alignment test results of OPLS-DA for the CO_2_-treated group.

Orthogonal partial least squares data analysis (OPLS-DA) was further applied to develop discriminative models that could differentiate the metabolites of the control group and CO_2_-treated group with different storage times. The results of the OPLS-DA models developed showed that R2X = 32.8%, R2Y = 99.7%, and Q2 = 95.2 in the control group and R2X = 35%, R2Y = 98.3%, and Q2 = 89.7% in the CO_2_-treated group, and the Q2 was greater than 0.5 based on the results of 500 tests of cross-validated alignments (Figure 5) with Q2s of 0.8988 and 0.8735 both being greater than 0.5, indicating that these models are reliable, and different treatments and storage time have significant effects on raw milk metabolites.

#### 2.4.3. Differential Metabolites (DMs)

**Figure 6:**
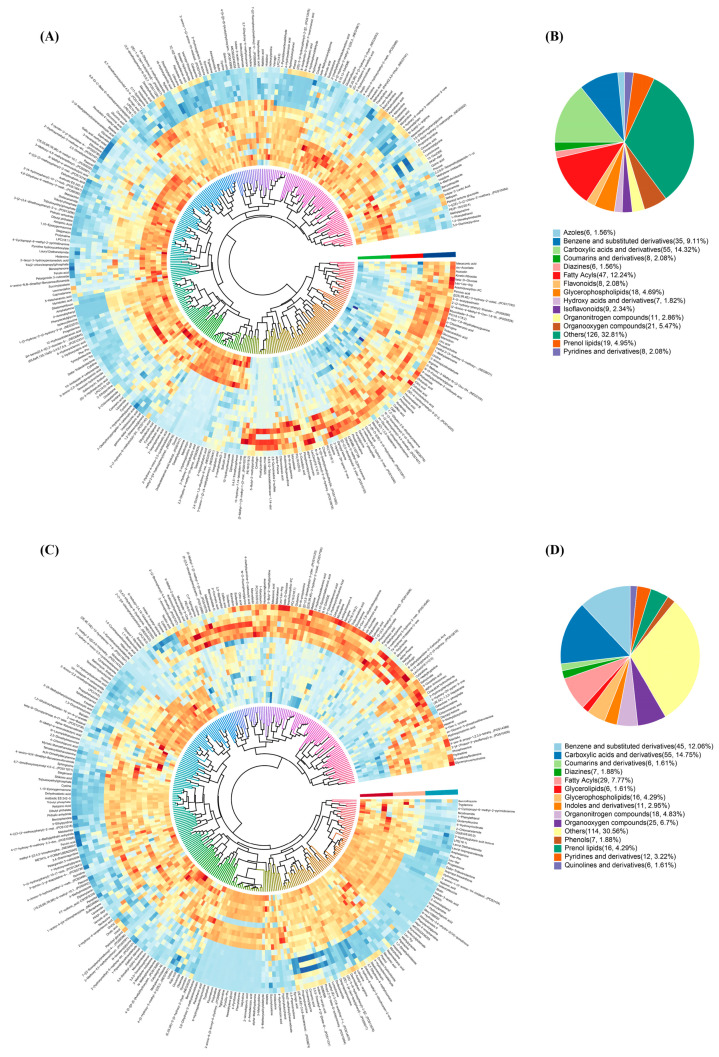
(**A**) Heatmap of DMs in the control group. (**B**) Heat map of DMs in the CO_2_-treated group. (**C**) Classification loop diagram (Ontology) of DMs in the control group. (**D**) Classification loop diagram (Ontology) of DMs in the CO_2_-treated group.

OPLS-DA analysis was used to obtain the variable importance for the projection (VIP) of metabolites to measure the strength of the influence of metabolite expression patterns on the categorical discrimination of each group of samples and the ability to explain, so as to assist in the screening of the marker metabolites (VIP > 1.0 was used as the screening criteria). DMs were indicated by VIP > 1 and *p* < 0.05. As shown in Figure 6A,C, 402 and 377 DMs were identified in the control and CO_2_-treated groups, respectively.

The structure and function of the metabolites and the DMs in each comparison group were categorized and counted by the class and ontology substance classification method of HMDB. As shown in Figure 6B,D, the main DMs in the raw milk were carboxylic acids and derivatives (A-14.32%, D-14.75%), fatty acyls (A-12.24%, D-7.77%), benzene and substituted derivatives (A-9.11%, D-12.06%), organooxygen compounds (A-5.47%, D-6.7%), and so on. Notably, the class classification of metabolites in the control and CO_2_-treated groups showed significant differences, mainly in the increase of 10 benzene and substituted derivatives and the decrease of 18 fatty acyl groups in the CO_2_-treated group.

### 2.5. Combined Microbiomics and Metabolomics Analysis

**Figure 7:**
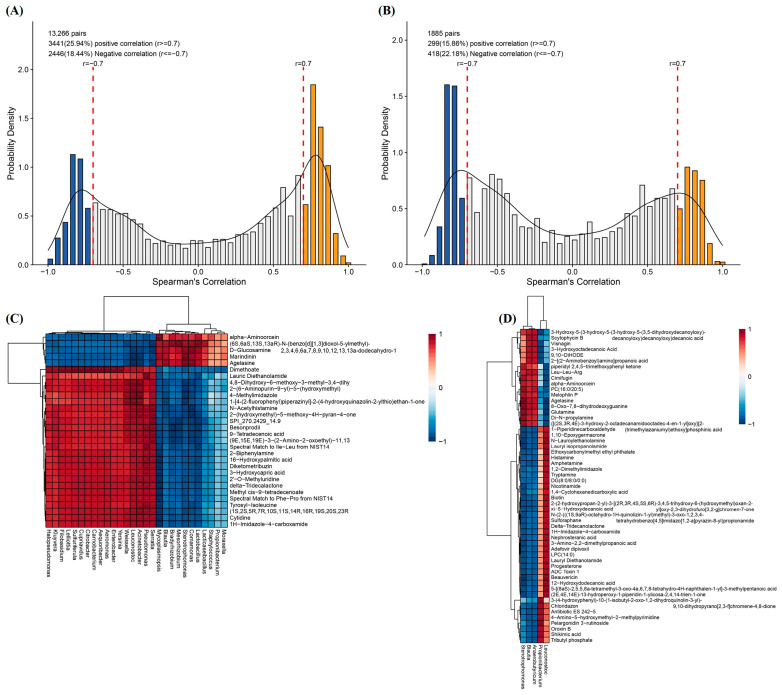
(**A**) Histogram of the distribution of microbiomics and metabolomics correlation coefficients (r) in the control group. (**B**) Histogram of the distribution of microbiomics and metabolomics correlation coefficients (r) in the CO_2_-treated group. The horizontal coordinate is the distribution of r between the two histologies, and the vertical coordinate is the density of the distribution of the corresponding r. r ≥ 0.7 or ≤−0.7 indicates that the correlation is very strong, i.e., the blue negative correlation and the yellow positive correlation part of the plot. (**C**) Hierarchical clustering heat map of microbial and metabolite correlations in the control group (Top 28). (**D**) Hierarchical clustering heatmap of microbial and metabolite correlations in the CO_2_-treated group. Rows indicate metabolites and columns indicate genera. *** denotes correlation test *p* < 0.001, ** denotes correlation test *p* < 0.01, * denotes correlation test *p* < 0.05.

Correlation analysis and elemental screening were performed to investigate the relationship between key microbial community structures and metabolite level parameters. As shown in Figure 7A,B, some microbes with metabolite changes were identified. Notably, the control microbial community showed a stronger positive correlation with metabolites, indicating that CO_2_ treatment was effective in reducing the metabolic activities of the raw milk system. The relationship between microbes and metabolites was shown by correlation hierarchical clustering heatmaps (Figure 7C,D). In the control group, the top 28 microorganisms (*Mycoplasmopsis*, *Aequoribacter*, *Aeromonas*, *Carnobacterium*, *Citrobacter*, *Cupriavidus*, etc.) and metabolites (delta-tridecalactone, cytidine, alpha-aminoorcein, D-glucosamine, 3-hydroxycapric acid, etc.) were correlated. Only five microorganisms, *Stenotrophomonas*, *Anaerobutyricum*, *Blautia*, *Leuconostoc* and *Propionibacterium* were correlated with the metabolites in the CO_2_-treated group compared to the control group.

### 2.6. Metabolic Pathway of Microbiomics and Metabolomics

**Figure 8:**
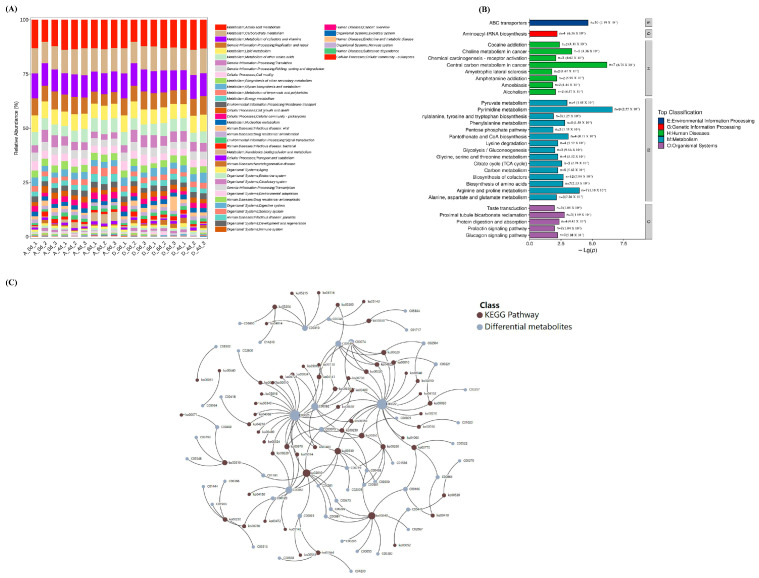
Microbiomics and untargeted metabolomics KEGG enrichment pathway analysis. (**A**) Microbiomics and identification of KEGG secondary metabolic pathway composition bar graphs for each sample. The vertical coordinates represent the relative abundance of each functional taxon; the longer the bar, the higher the relative abundance of that functional taxon in the corresponding sample. (**B**) Comparison group A-6d and D-6d KEGG pathway category bar graphs. G is Genetic Information Processing, M is Metabolism, E is Environmental Information Processing, o is Organismal Systems, H is Human Diseases. (**C**) Interaction maps of KEGG-enriched pathway-regulated metabolite networks shared by microbiomics and non-targeted metabolomics.

Based on Illumina NovaSeq/HiSeq high-throughput sequencing platform, the whole genome shotgun (WGS) strategy was used to extract the total DNA of the macro-genome of the obtained colonies, or the cDNA double strand of the macro-transcriptome synthesized by using mRNA as a template. The coding regions of prokaryotic microbial and macrogenomic gene sequences were predicted to obtain the corresponding gene sequence files, protein sequence files for protein sequence species and functional annotation. As shown in Figure 8A and Table S1, CO_2_ treatment significantly reduced metabolic processes, such as metabolism (limonene and pinene degradation, energy metabolism, xenobiotics biodegradation and metabolism, C5-branched dibasic acid metabolism, β-alanine metabolism, monobactam biosynthesis, caprolactam and styrene degradation), cellular processes (bacterial chemotaxis and biofilm formation—pseudomonas aeruginosa), environmental information processing (ABC transporters), and genetic information processing (sulfur relay system).

The metabolic activities of environmental information processing (ABC transporters), metabolism (biosynthesis of cofactors, pyrimidine metabolism, arginine and proline metabolism, biosynthesis of amino acids, carbon metabolism, phenylalanine metabolism, 2-oxocarboxylic acid metabolism, and pantothenate and CoA biosynthesis), and organismal systems (protein digestion and absorption) were significantly stronger in the control group than in the CO_2_-treated group, and microbial metabolism and cellular processes altered raw material metabolites (Figure 8B and Table S1). As shown in Table 1 of Figure 8C, shared metabolic pathways identified by the macrogenome and metabolome mainly included cellular processes, environmental information processing, genetic information processing, metabolism and organismal systems. A total of 61 shared metabolic pathways involving 53 DMs were identified. Among them, ABC transporters, pyrimidine metabolism, arginine and proline metabolism and phenylalanine metabolism metabolic processes had the most significant effects on metabolites, involving metabolites, such as pyruvic acid (C00022), glutamic acid (C00025), 5′-CMP (C00055), arginine (C00062), 2-propenoic acid (C00074), and phenylalanine (C00079).

## 3. Discussion

In the present study, *Stenotrophomonas maltophilia*, *Lactococcus lactis* and *Chryseobacterium* sp. *NEB161* were found to be the dominant strains of raw milk, with a high percentage of 81.7%. The cold storage process altered the microbial colony diversity of raw milk, and the dominant strains at 6 days were *Acinetobacter guillouiae*, *Pseudomonas fluorescens*, *Serratia liquefaciens*, *Pseudomonas simiae* and *Leuconostoc mesenteroides*, with a percentage of 56.2%. These results also verified that the microbial community is a key factor in the spoilage of raw milk. Therefore, it is necessary to use appropriate techniques to inhibit the growth and multiplication of microorganisms in raw milk. Research has shown that adding CO_2_ to food reduced the rate of food spoilage and the growth of disease-causing microorganisms [18]. It was found that CO_2_ treatment significantly inhibited the propagation of the dominant colonies, *Acinetobacter guillouiae*, *Pseudomonas fluorescens*, *Serratia liquefaciens* and *Pseudomonas simiae* as compared with the control.The percentage in the CO_2_-treated group was only 0.2%. CO_2_ treatment significantly improved the storage period of raw milk, indicating that CO_2_ improved the quality of raw milk by suppressing microbial levels and diversity.

Through microbiomics analysis, CO_2_ was found to prolong the storage period of raw milk by inhibiting microbial reproduction, but the exact mechanism of its antimicrobial effect is still unclear [13]. CO_2_ is highly soluble in water and lipids, and can form carbonic acid in aqueous solutions, which can lead to changes in pH in the suspension medium, and a decrease in pH inhibits the growth and metabolism of certain microorganisms [19]. The current study revealed the reduction in the pH of raw milk treated with CO_2_. Another view is that oxygen is a necessary electron acceptor in the metabolism of aerobic bacteria and is a specific electron acceptor for parthenogenetic anaerobes. Supplementation of raw milk with CO_2_ reduced the oxygen content of the system, which in turn inhibited bacterial metabolism and reduced growth rates [20]. CO_2_ treatment significantly decreased the levels of *Acinetobacter guillouiae*, *Pseudomonas fluorescens*, *Serratia liquefaciens* and *Pseudomonas simiae*, which inhibited Gram-negative aerobic bacteria, suggesting that CO_2_ inhibited the growth and reproduction of aerobic microorganisms and prolonged the shelf life of raw milk by reducing the oxygen content of the system.

Microorganisms in raw milk systems can utilize the nutrients in milk for growth and reproduction while breaking down substances, such as proteins, fats and lactose. For example, the pH of milk decreases when lactose is broken down to lactic acid [21,22]. Proteins are broken down, producing toxic substances, such as indole, sulfur and fecal deodorant, as well as foul odors. To be precise, the growth and reproduction of microorganisms is the metabolic process which leads to changes in the metabolites of the raw milk system through a variety of metabolic processes reducing the stability of the raw milk system and even spoilage [22,23]. It is worth noting that the microbial effects on metabolites are mainly metabolic, including pyrimidine metabolism, arginine and proline metabolism, phenylalanine metabolism, aminoacyl tRNA biosynthesis, glycine, serine and threonine metabolism, lysine degradation, pantothenate and coenzyme A biosynthesis, and pyruvate metabolism (Table 1).

Pyrimidine metabolism refers to the process of pyrimidine nucleotide synthesis and degradation in the cell and is an important component of nucleic acid synthesis. Through pyrimidine metabolism, cells are able to synthesize and maintain sufficient levels of pyrimidine nucleotides to support DNA and RNA synthesis and the stability of genetic material [24,25]. Microbial reproduction requires the replication of genetic material, and reduced levels of metabolites, such as cytidylic acid, orotate and uridine, inhibit microbial growth and reproduction. In addition, pyrimidine nucleotide degradation produces uracil, which in turn is converted to β-aminobutyric acid, which is involved in amino acid and energy metabolic pathways. The inhibition of energy production results from reduced levels of orotate [26]. The accumulation of N-carbamoyl-L-aspartate, a key compound in the metabolism of alanine, aspartic acid and glutamic acid suggested that the processes involved in the synthesis of amino acids and the metabolism of other nitrogen compounds were inhibited.

Arginine and proline metabolism can generate nitric oxide and polyamine compounds, which play important roles in physiological processes, such as cell signaling, immunomodulation and blood flow regulation [27]. In addition, proline metabolism produces polyamines and cofactors required for the post-translational modification of proteins, which play key roles in cell growth and development [28]. Phenylalanine metabolism produces tyrosine and other important bioactive substances which play important roles in neurotransmitter, hormone and pigment synthesis. Glycine, serine and threonine metabolism involves a variety of enzymes and intermediates that have multiple effects on metabolites. For example, glycine metabolism produces alanine, which is involved in glucose and fatty acid metabolic pathways. Serine metabolism produces tryptophan. Threonine metabolism produces threonine and methionine, the compounds that play important roles in methionine metabolism and thionine metabolism [29]. Amino acid metabolic processes are attenuated, but accumulation of betaine occurs in the glycerophospholipid metabolic pathway of glycine, serine, and threonine metabolism, which may be due to enhanced upstream glycerophospholipid metabolic activity. Lysine degradation generates intermediates, such as malondialdehyde, acetaldehyde and aminobutyric acid, which can be further involved in oxidation that can alter the stability of the raw milk system [30]. Reduction in glutarate and N6, N6, N6-trimethyl-L-lysine levels were found in the lysine degradation pathway. It was found that CO_2_ treatment significantly inhibited amino acid metabolism and reduced microbial biological activity and acid accumulation. In addition, metabolic processes, such as carbohydrate metabolism, lipid metabolism, cell growth and death, and energy metabolism were also inhibited. Overall, CO_2_ treatment effectively reduced the metabolism of microorganisms and thus inhibited their growth and reproduction.

## 4. Materials and Methods

### 4.1. Sample Collection

Fresh raw milk [the somatic cells (<2 × 10^5^/mL) were counted using a flow cell fluorescence counter (Counters II FL, Thermo Fisher, Waltham, MA, USA), and the TBC (<10^3^ CFU/mL) was counted using the spread plate method] was collected aseptically from milk tanks at Helan Mountain Dairy Farm, Ningxia, Yinchuan, China in June 2022, refrigerated at 4 °C and transported to the laboratory within one hour. Then, 0.6 g solid CO_2_ (dry ice, food grade, Yinchuan, China) was added to 500 mL PET bottles (Servicebio, Wuhan, China) containing 300 mL of raw milk to achieve a CO_2_ concentration of 2000 ppm in a sterile room immediately [31]. Raw milk without CO_2_ treatment was used as a control group. The control group and the treated group were divided into 17 and 7 equal portions of 100 mL in sterile sealed PET bottles, respectively, and stored at 4 °C. One aliquot was used for analysis each day. The day of sample collection was defined as day 0.

### 4.2. Bacterial Growth Studies

The TBC was measured by the spread plate method. Briefly, 1 mL sample was taken and serially diluted 10 times with 0.85% sterile saline. Then, the 200 μL dilutions were spread onto plate count agar (PCA) and cultured at 37 °C for 48 h. Analysis of a sample was terminated when it was spoiled, i.e., the TBC reached a threshold of 6 lg (cfu/mL) [32].

### 4.3. pH Measurement

Raw milk pH was determined using a pH acidimeter (PHS-3C, Shanghai Yidian Scientific Instrument Co., Ltd., Shanghai, China) with a three-point calibration standard prior to testing. CO_2_-treated samples were degassed with agitation to eliminate the effect of CO_2_ on the sample [33], and then the pH was measured again.

### 4.4. Colour Measurement

Briefly, 30 mL of the degassed sample were added into a transparent container, and a hand-held colorimeter (CR-400, Konica Minoita, Tokyo, Japan) was used to measure the parameters L*, a*, and b* of the milk sample. L* denoted the brightness, a* denoted the red and green values, and b* denoted the yellow and blue values. Based on the obtained values of L*, a*, and b*, the ΔE between the control group and the CO_2_-treated group was calculated, by the following formula: ΔE = [(ΔL*)^2^ + (Δa*)^2^ + (Δb*)^2^]^1/2^. Where ΔL*, Δa* and Δb* are the differences between the control group and the CO_2_-treated group for L*, a* and b*, respectively.

### 4.5. Viscosity Measurement

Referring to the method described by Ning et al. [34], the specific viscosity of the degassed raw milk was determined using an Ubbelohde viscometer (Shanghai Shenyi Glass Products Co., Ltd., Shanghai, China). The specific viscosity was calculated by the ratio of the outflow time of a certain amount of liquid to the outflow time of the control liquid (deionized water).

### 4.6. Zeta Potential Measurement

The zeta potential values of casein micelles were determined using a nanoparticle size potential analyzer (Malvern, UK). The degassed raw milk samples were diluted 2-fold with deionized water, and the dilutions were used to determine the zeta potential at room temperature. The test mode was automatic with 3 replicates for each sample.

### 4.7. Metagenomics Analysis of Raw Milk

#### 4.7.1. Raw Milk DNA Extraction and Processing

After centrifugation of raw cow’s milk, all microbial DNA in raw cow’s milk was extracted by DNeasy PowerFood Microbial DNA Isolation Kit (QIAGEN, Dusseldorf, Germany). DNA integrity was determined by 1.2% agarose gel and gel Doc EZ imager (Bio-Rad, Hercules, CA, USA), and DNA concentration was measured by Qubit 4.0 Fluorescence Quantification Instrument (Invitrogen, Carlsbad, CA, USA). Qubit™ dsDNA HS Assay Kit (Invitrogen, Carlsbad, CA, USA) was used to accurately quantify the concentration of the genome and determine the total amount of DNA required for library construction. The DNA was fragmented using Covaris 220 ultrasonic crusher (Woburn, MA, USA), concentrated and recovered, and then purified and its concentration determined using Qubit 4.0.

#### 4.7.2. Library Construction and Sequencing

Library construction was carried out according to the instructions of the library construction kit (ONT, Oxford, UK) for Hieff NGS^®^ high-throughput sequencing platform. Briefly, the purified DNA was subjected to end modification using NEBNext FFPE DNA Repair kit (New England Biolabs, Ipswich, MA, USA), junction ligation and magnetic bead sorting to purify the ligated product, and the purified product was subjected to PCR amplification and enrichment, and the PCR amplification product was purified, and the size of library was determined by 2% agarose gel electrophoresis, and the concentration of library was measured by Qubit 4.0, and the qualified library was used. After the libraries were qualified, the Illumina NovaSeq platform (Illumina, San Diego, CA, USA) was used to analyze the libraries by end-pair sequencing.

#### 4.7.3. Data Analysis

In order to ensure the quality of information, the raw data were filtered, and the clean data were obtained by removing the sequences with junctions and low-quality sequences. After removing the junctions in the reads, global cropping and window quality cropping, and the host contamination, the quality of the sequenced raw data were evaluated by Fastp-0.36, and the validated reads were analyzed statistically after the data filtering.

### 4.8. Metabolomics Analysis of Raw Milk

#### 4.8.1. Metabolite Extraction

Raw cow’s milk was vortexed at a low speed, 100 μL of sample was pipetted into a centrifuge tube, then 400 μL of pre-cooled pure methanol solution was added, vortexed and mixed, then sonicated in an ice bath for 20 min, and then allowed to stand at −20 °C for 1 h. Frozen samples were centrifuged at 16,000× *g*, 4 °C for 20 min, and the supernatant was dried in a high-speed vacuum concentrator centrifuge. The supernatant was dried in a high-speed vacuum concentration centrifuge. The dried sample was raw milk metabolites, and 100 μL of methanol-water solution (1:1, *v*/*v*) were added to dissolve the metabolites for spectrometry, and then centrifuged at 20,000× *g*, at 4 °C for 15 min, and then the supernatant was taken as the sample for analysis.

#### 4.8.2. LC-MS/MS Analysis

##### Chromatographic Separation

The samples were analyzed in an autosampler at 4 °C. The samples were separated on a SHIMADZU-LC30 ultra-high-performance liquid chromatography (UHPLC) system (Shimadzu, Kyoto, Japan) using an ACQUITY UPLC^®^ HSS T3 column (2.1 × 100 mm, 1.8 µm) (Waters, Milford, MA, USA). The flow rate was 0.3 mL/min at 40 °C with an injection volume of 4 μL. The chromatographic mobile phases were A: 0.1% formic acid aqueous solution, and B: acetonitrile. The chromatographic gradient elution program was as follows: from 0 to 2 min, 100% A; from 2 to 6 min, A varied linearly from 100% to 52%; from 6 to 10 min, A varied linearly from 52% to 0%; from 10 to 12 min, A was maintained at 0%; from 12 to 12.1 min, A was maintained at 0%; from 12 to 12.1 min, A was maintained at 0%; and from 12 to 12.1 min, A was maintained at 0%. 0%; from 12 to 12.1 min, A varied linearly from 0% to 100%, and from 12.1 to 15 min, A was maintained at 100%.

##### Mass Spectrometry Acquisition

Each sample was collected by electrospray ionization (ESI) in positive (+) and negative (−) modes. The samples were separated by UPLC and analyzed by mass spectrometry using a QE Plus mass spectrometer (Thermo Scientific, Waltham, MA, USA). The ionization conditions were as follows: spray voltage: 3.8 kv (+) and 3.2 kv (−); capillary temperature: 320 (±); spray voltage: 3.8 kv (+) and 3.2 kv (−); capillary temperature: 320 (±); sheath gas: 30 (±); aux gas: 5 (±); probe heater temp: 350 (±); S-lens RF level: 50.

#### 4.8.3. Data Pre-Processing

The raw data were subjected to peak alignment, retention time correction and peak area extraction using MSDIAL software (ver 4.18). The structural identification of metabolites was performed by exact mass number matching (mass tolerance < 10 ppm) and secondary spectra matching (mass tolerance < 0.01 Da), and the public databases, such as HMDB, MassBank, GNPS, etc., as well as the self-constructed metabolite standard library of Dubai Spectrum (BP-DB), were searched. Ion peaks with >50% missing values were removed from the extracted data and were not included in the subsequent statistical analysis.

### 4.9. Statistical Analysis

To identify the perturbed biological pathways, the DM data were subjected to KEGG pathway analysis using KEGG database (http://www.kegg.jp (accessed on 18 May 2023)). KEGG enrichment analyses were carried out with the Fisher’s exact test, and an FDR correction was applied to account for multiple testing. Enriched KEGG pathways were considered statistically significant at the *p* < 0.05 level. All physicochemical data were analyzed by one way ANOVA and *t*-test using SPSS 25 program (SPSS Inc., Chicago, NY, USA), and significant difference was considered at *p* < 0.05. Plotting was done using Origin 2023 (Origin Lab., Northampton, MA, USA).

## 5. Conclusions

It was found that CO_2_ at 2000 ppm extended the shelf life of raw milk from 6 to 16 days without adversely affecting emulsion stability and color, providing a theoretical basis for its practical application. CO_2_ mainly inhibited Gram-negative aerobic bacteria, such as *Acinetobacter guillouiae*, *Pseudomonas fluorescens*, *Serratia liquefaciens* and *Pseudomonas simiae*, and their growth and multiplication accelerated ABC transporters, pyrimidine metabolism, amino acid metabolism, carbohydrate and lipid metabolism, and metabolism-produced metabolites can lead to raw milk quality deterioration. Therefore, the use of CO_2_ is important for the storage, preservation and quality control of raw milk, and can be explored for implementation in dairy farms.

## Figures and Tables

**Table 1:** Macrogenomics screened proteins corresponding to KO abundance and metabolomics annotated KEGG shared pathway analysis.

No.	ID	ID	Description	Count	KEGG ID	Top Class
1	ko02010	bta02010	ABC transporters	10	C00719/C00062/C00025/C00120/C00093/C00299/C00475/C00881/C00079/C01181	Metabolism
2	ko00240	bta00240	Pyrimidine metabolism	9	C00055/C00380/C00299/C00475/C00881/C00106/C00438/C02067/C00295	Environmental Information Processing
3	ko00330	bta00330	Arginine and proline metabolism	7	C00062/C00581/C02305/C00025/C04281/C00022/C00300	Metabolism
4	ko00360	bta00360	Phenylalanine metabolism	5	C00082/C01586/C00805/C00079/C00022	Metabolism
5	ko00770	bta00770	Pantothenate and CoA biosynthesis	4	C00864/C00522/C00106/C00022	Metabolism
6	ko00620	bta00620	Pyruvate metabolism	4	C02504/C00074/C00149/C00022	Metabolism
7	ko00260	bta00260	Glycine, serine and threonine metabolism	4	C00719/C00581/C00022/C00300	Organismal Systems
8	ko00310	bta00310	Lysine degradation	4	C03793/C00489/C01181/C05548	Metabolism
9	ko00970	bta00970	Aminoacyl-tRNA biosynthesis	4	C00062/C00025/C00082/C00079	Human Diseases
10	ko04141	bta04964	Proximal tubule bicarbonate reclamation	3	C00074/C00025/C00149	Metabolism
11	ko00020	bta00020	Citrate cycle (TCA cycle)	3	C00074/C00149/C00022	Metabolism
12	ko04922	bta04922	Glucagon signaling pathway	3	C00074/C00149/C00022	Organismal Systems
13	ko00250	bta00250	Alanine, aspartate and glutamate metabolism	3	C00025/C00438/C00022	Genetic Information Processing
14	ko05204	bta05207	Chemical carcinogenesis—receptor activation	3	C14240/C03690/C00410	Metabolism
15	ko00010	bta00010	Glycolysis/Gluconeogenesis	3	C00074/C00221/C00022	Human Diseases
16	ko00400	bta00400	Phenylalanine, tyrosine and tryptophan biosynthesis	3	C00074/C00082/C00079	Human Diseases
17	ko00030	bta00030	Pentose phosphate pathway	3	C00257/C00221/C00022	Metabolism
18	ko00564	bta00564	Glycerophospholipid metabolism	3	C00588/C00093/C04230	Metabolism
19	ko00630	bta00630	Glyoxylate and dicarboxylate metabolism	3	C00025/C00149/C00022	Metabolism
20	ko00380	bta00380	Tryptophan metabolism	3	C01717/C05834/C00328	Human Diseases
21	ko00230	bta00230	Purine metabolism	3	C00366/C01444/C05513	Metabolism
22	ko00460	bta05030	Cocaine addiction	2	C00025/C00082	Metabolism
23	ko05034	bta05034	Alcoholism	2	C00025/C00082	Metabolism
24	ko05014	bta05014	Amyotrophic lateral sclerosis	2	C00062/C00025	Environmental Information Processing
25	ko00220	bta00220	Arginine biosynthesis	2	C00062/C00025	Cellular Processes
26	ko00290	bta00290	Valine, leucine and isoleucine biosynthesis	2	C02504/C00022	Organismal Systems
27	ko00780	bta00780	Biotin metabolism	2	C00120/C01909	Metabolism
28	ko04216	bta04216	Ferroptosis	2	C00025/C00418	Metabolism
29	ko05200	bta05200	Pathways in cancer	2	C00410/C00149	Cellular Processes
30	ko00730	bta00730	Thiamine metabolism	2	C00082/C00022	Environmental Information Processing
31	ko00410	bta00410	beta-Alanine metabolism	2	C00864/C00106	Human Diseases
32	ko00650	bta00650	Butanoate metabolism	2	C00025/C00022	Metabolism
33	ko00760	bta00760	Nicotinate and nicotinamide metabolism	2	C01020/C00022	Metabolism
34	ko00350	bta00350	Tyrosine metabolism	2	C00082/C00022	Human Diseases
35	ko00520	bta00520	Amino sugar and nucleotide sugar metabolism	2	C00270/C00446	Human Diseases
36	ko00860	bta00860	Porphyrin and chlorophyll metabolism	2	C00025/C02800	Human Diseases
37	ko04150	bta04150	mTOR signaling pathway	1	C00062	Metabolism
38	ko04114	bta04114	Oocyte meiosis	1	C00410	Metabolism
39	ko04914	bta04914	Progesterone-mediated oocyte maturation	1	C00410	Metabolism
40	ko04068	bta04068	FoxO signaling pathway	1	C00025	Human Diseases
41	ko05142	bta05142	Chagas disease	1	C00062	Metabolism
42	ko05016	bta05016	Huntington disease	1	C00025	Organismal Systems
43	ko05143	bta05143	African trypanosomiasis	1	C00328	Metabolism
44	ko00472	bta00472	D-Arginine and D-ornithine metabolism	1	C00062	Environmental Information Processing
45	ko05215	bta05215	Prostate cancer	1	C00410	Metabolism
46	ko04721	bta04721	Synaptic vesicle cycle	1	C00025	Environmental Information Processing
47	ko04066	bta04066	HIF-1 signaling pathway	1	C00022	Metabolism
48	ko00910	bta00910	Nitrogen metabolism	1	C00025	Human Diseases
49	ko04152	bta04152	AMPK signaling pathway	1	C00022	Metabolism
50	ko05012	bta05012	Parkinson disease	1	C00082	Metabolism
51	ko00480	bta00480	Glutathione metabolism	1	C00025	Metabolism
52	ko00561	bta00561	Glycerolipid metabolism	1	C00093	Metabolism
53	ko00640	bta00640	Propanoate metabolism	1	C05984	Metabolism
54	ko00052	bta00052	Galactose metabolism	1	C00446	Metabolism
55	ko00340	bta00340	Histidine metabolism	1	C00025	Metabolism
56	ko00071	bta00071	Fatty acid degradation	1	C00489	Environmental Information Processing
57	ko00524	bta04080	Neuroactive ligand-receptor interaction	1	C00025	Metabolism
58	ko00061	bta00061	Fatty acid biosynthesis	1	C08362	Metabolism
59	ko00040	bta00040	Pentose and glucuronate interconversions	1	C00022	Metabolism
60	ko00270	bta00270	Cysteine and methionine metabolism	1	C00022	Metabolism
61	ko00130	bta00130	Ubiquinone and other terpenoid-quinone biosynthesis	1	C00082	Metabolism

## Data Availability

Data are contained within the article and supplementary materials.

## Supplementary Materials

The following supporting information can be downloaded at: https://www.mdpi.com/article/10.3390/molecules29020329/s1.
